# Supplementary material for: Expression of CD44 in Leukocyte Subpopulations in Patients with Inflammatory Bowel Diseases
Source: Diagnostics (Basel). 2022 Aug 20;12(8):2014. doi: 10.3390/diagnostics12082014 (PMC9407096; doi:10.3390/diagnostics12082014)
Supplement: Supplementary file 1 [file diagnostics-12-02014-s001.zip › diagnostics-1850040-supplementary.pdf]

Expression of CD44 in monocyte and lymphocyte subpopulations in patients with inflammatory bowel diseases

Ivana Franić[1], Nikolina Režić Mužinić[2]*, Anita Markotić[2], Piero Marin Živković[3], Marino Vilović[4], Doris Rušić[5], and Joško Božić[4]

Table S1. Basic characteristics of IBD and control group.

Parameter	IBD (n = 46)	Control (n = 48)	*p*
Age	40.97 ± 12.65	39.04 ± 12.93	0.456*
Male gender	26 (56.5%)	34 (70.8%)	0.149**
Body weight (kg)	75.054 ± 15.47	83.02 ± 13.01	0.008*
Body height (cm)	176 ± 9.52	181.14 ± 9.54	0.0117*
BMI	24.12 ± 4.27	25.2 ± 2.78	0.15*
Active smoking	13 (28.2%)	10 (20.8%)	0.402**

Basic parametric data are presented as mean ± standard deviation for parametric data, categorical data are presented as number (percentage). IBD: inflammatory bowel disease; BMI: body mass index; *Student t-test for independent samples. ** Chi-square test.

Table S2. Comparison of basic anthropometric and disease characteristics between Ulcerative Colitis and Crohn's Disease.

Parameter	UC (n = 18)	Crohn (n = 28)	*p*
Age	42 ± 12.3	40.32 ± 13.04	0.66*
Male gender	10 (55.5%)	16 (57.1%)	0.577**
Body weight (kg)	79.78 ± 13.78	72 ± 15.98	0.097*
Body height (cm)	176.80 ± 7.52	176.6 ± 10.72	0.67*
BMI	25.55 ± 4.30	23.211 ± +4.06	0.069*
Active smoking	1 (5.5%)	12 (66.6%)	0.006**
SES CD	/	13.67 ± 11.17	
UCIS	4.38 ± 2.91	/	

Basic parametric data are presented as mean ± standard deviation, categorical data are presented as number (percentage). BMI: body mass index; SES-CD: simple endoscopic score for Crohn's disease; UCEIS: ulcerative colitis endoscopic index of severity; UC: Ulcerative Colitis * Student t-test for independent samples. ** Chi-square test. *** Mann–Whitney U test.

Table S3. Comparison of selected laboratory characteristics of Ulcerative Colitis and Crohn's Disease.

Parameter	UC (n = 18)	Crohn (n = 28)	*p*
WBC ($x10^9$/L)	5.68 ± 1.45	7.6 ± 2.92	0.013*
RBC ($x10^{12}$/L)	4.619 ± 0.76	4.64 ± 0.49	0.892*
Platelets ($x10^9$/L)	232 ± 72.39	283 ± 87.99	0.054*
Albumins (g/L)	39.59 ± 3.89	37.14 ± 6.17	0.14*
ESR (mm/h)	15.94 ± 16.22	16.82 ± 19.49	0.875*
Hb (g/L)	137.22 ± 23.14	134.82 ± 18.52	0.699*
Hct (L/L)	0.416 ± 0.065	0.41 ± 0.049	0.762*
LDH (mmol/L)	168 ± 34.12	161.22 ± 48.41	0.57*
ALP (U/L)	73.75 ± 48.29	79.66 ± 39.42	0.55*
hs-CRP (mg/l)	2.52 ± 3.91	14.77 ± 30.96	0.103*
FC (mg/kg)	356.05 ± 480.49	753.32 ± 1314.35	0.226*

Data are presented as mean ± standard deviation. UC: Ulcerative Colitis; WBC – white blood cells; RBC – red blood cells; ESR – erythrocyte sedimentation rate; Hb – hemoglobin; Hct – hematocrit; LDH – lactate dehydrogenase; ALP – alkaline phosphatase; hs-CRP – high sensitivity C-reactive protein; FC – fecal calprotectin. *Student t-test for independent samples.

Table S4. Comparison of selected disease characteristics between Crohn's Disease subgroups according to biologic therapy.

Parameter	Biological therapy (n = 18)	Non-biological therapy (n = 10)	*p*
Age	41.44 ± 13.87	38.3 ± 11.83	0.0551*
Male gender	11 (61.1%)	5 (50%)	0.43**
Body weight (kg)	74.6 ± 14.06	67.3 ± 18.81	0.251*
Body height (cm)	175 ± 10.35	175 ± 11.92	0.814*
BMI	24 ± 3.64	21.44 ± 4.36	0.085*
Active smoking	8 (44.4%)	2 (20%)	0.076**
SES CD	11.12 ± 9.69	18.24 ± 12.69	0.108*

Basic parametric data are presented as mean ± standard deviation, categorical data are presented as number (percentage). BMI: body mass index; SES-CD: simple endoscopic score for Crohn's disease; * Student t-test for independent samples. ** Chi-square test. *** Mann–Whitney U test.

Table S5. Comparison of selected laboratory characteristics of Crohn's Disease subgroups according to biologic therapy.

Parameter	Biological therapy (n = 18)	Non-biological therapy (n = 10)	*p*
WBC (x10^9/L)	8.2 ± 3.3	6.51 ± 1.73	0.144*
RBC (x10^{12}/L)	4.76 ± 0.63	4.41 ± 0.91	0.653*
Platelets (x10^9/L)	261 ± 59.4	323 ± 117.4	0.072*
Albumins	38.66 ± 4.1	34.3 ± 8.33	0.08*
ESR (mm/h)	11.22 ± 9.43	26.9 ± 28.2	0.038*
Hb (g/L)	139 ± 12.78	126 ± 24.45	0.072*
Hct (L/L)	0.432 ± 0.045	0.396 ± 0.084	0.127*
LDH (mmol/L)	164.5 ± 26.17	174.7 ± 46.1	0.459*
ALP (U/L)	63.55 ± 16	92.1 ± 77	0.136*
FC (mg/kg)	265.11 ± 299	1632 ± 1917	0.005*
hs-CRP (mg/l)	8.88 ± 24.09	25.37 ± 39.81	0.181*

Data are presented as mean ± standard deviation. WBC – white blood cells; RBC – red blood cells; ESR – erythrocyte sedimentation rate; Hb – hemoglobin; Hct – hematocrit; LDH – lactate dehydrogenase; ALP – alkaline phosphatase; FC – fecal calprotectin; hs-CRP – high sensitivity C-reactive protein. *Student t-test for independent samples.

Table S6. Comparison of selected disease characteristics between Ulcerative colitis subgroups according to biologic therapy.

Parameter	Biological therapy (n = 10)	Non-biological therapy (n = 8)	*p*
Age	43.4 ± 14.06	40.25 ± 10.36	0.6*
Male gender	6 (60%)	4 (50%)	0.52**
Body weight (kg)	84.5 ± 12.176	73.87 ± 14.09	0.105*
Body height (cm)	176 ±.11	176 ± 8.51	0.94*
BMI	26.98 ± 3.53	23.75 ± 4.73	0.116*
Active smoking	0 (0%)	1 (12.5%)	0.444**
USEIS	4.5 ± 2.63	4.25 ± 3.41	0.862*

Basic parametric data are presented as mean ± standard deviation, non-parametric data are presented as median (interquartile range) and categorical data are presented as number (percentage). BMI: body mass index; UCEIS: ulcerative colitis endoscopic index of severity; * Student t-test for independent samples. ** Chi-square test. *** Mann–Whitney U test.

Table S7. Comparison of selected laboratory characteristics of Ulcerative colitis subgroups according to biologic therapy.

Parameter	Biological therapy (n = 10)	Non-biological therapy (n = 8)	*p*
WBC (x10^9/L)	6.2 ± 1.45	4.93 ± 1.067	0.048*
RBC (x10^{12}/L)	4.67 ± 0.435	4.58 ± 0.604	0.316*
Platelets (x10^9/L)	247 ± 85.15	214 ± 34.97	0.319*
Albumins	39.9 ± 1.59	39.2 ± 5.72	0.715*
ESR (mm/h)	12.4 ± 11.37	20.37 ± 20.79	0.314*
Hb (g/L)	141 ± 17.08	132 ± 29.62	0.44*
Hct (L/L)	0.42 ± 0.035	0.39 ± 0.067	0.263*
LDH (mmol/L)	176.2 ± 29.5	142.5 ± 62.05	0.147*
ALP (U/L)	73.8 ± 16.04	87 ± 57.7	0.496*
FC (mg/kg)	212.2 ± 328.5	535 ± 596	0.161*
hs-CRP (mg/l)	1.49 ± 1.52	3.81 ± 5.53	0.22*

Data are presented as mean ± standard deviation. WBC – white blood cells; RBC – red blood cells; ESR – erythrocyte sedimentation rate; Hb – hemoglobin; Hct – hematocrit; LDH – lactate dehydrogenase; ALP – alkaline phosphatase; FC – fecal calprotectin; hs-CRP – high sensitivity C-reactive protein. *Student t-test for independent samples.
